# Notice to Readers About the Cover

**DOI:** 10.3201/eid2912.AC2912

**Published:** 2023-12

**Authors:** Byron Breedlove

**Affiliations:** Centers for Disease Control and Prevention, Atlanta, Georgia, USA

**Keywords:** about the cover, art science connection, emerging infectious diseases, art and medicine, public health

**Figure:**
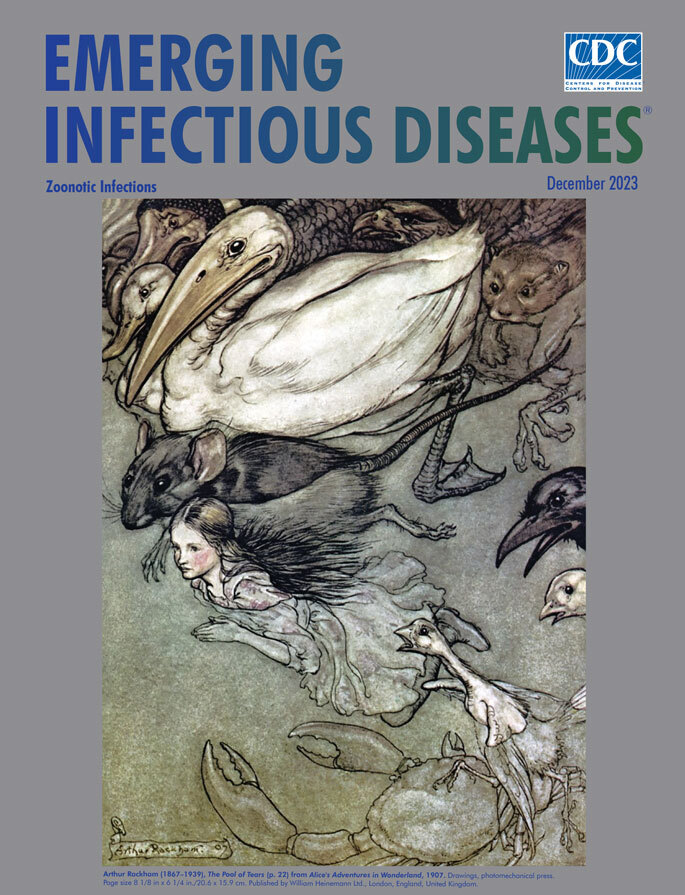
**Arthur Rackham (1867–1939), “The Pool of Tears” (p. 22), from *Alice's Adventures in Wonderland*, 1907.** Drawings, photomechanical press. Page size 8 1/8 in x 6 1/4 in/20.6 x 15.9 cm. Published by William Heinemann Ltd., London, United Kingdom. Public domain image available at https://www.gutenberg.org/files/28885/28885-h/28885-h.htm.

*Suggested citation for this article*: Breedlove B. Notice to readers about the cover. Emerg Infect Dis. 2023 Dec [*date cited*]. https://doi.org/10.3201/eid2912.AC2912

Appearing on this month’s cover is an image titled “The Pool of Tears.” The image comes from the 1907 edition of *Alice’s Adventures in Wonderland*, written by Lewis Carroll (his actual name was Charles Lutwidge Dodgson) and illustrated by the British artist Arthur Rackham.

Rather than following our usual practice of publishing a short essay to accompany the cover image, this month *Emerging Infectious Diseases* is instead using this space to make a call for About the Cover submissions from readers interested in the intersection of art and science, medical history, and biographical sketches. Those considering this invitation should first read the information on our website at About Cover Art (https://wwwnc.cdc.gov/eid/page/about-cover-art).

Cover essays are intended to relate science and the human condition, or how people perceive and cope with infection and illness. EID seeks for its cover images and their accompanying essays to evoke compassion for human suffering and to expand the science reader's literary or artistic scope, provide context related to the theme of each month’s issue, and link the art with public health and science. Although we want scientific information mentioned in about the cover essays to be accurate, these are not research articles.

 Each issue’s theme is typically decided and assigned two to ten months before the publication date. Typical themes include zoonotic infections, vectorborne infections, respiratory infections, emerging viruses or emerging pathogens, fungal infections, bacterial infections, parasitic diseases, or some variation of those general themes. EID also publishes issues with themes related to specific diseases or health topics.

All genres of art from all cultures, historical periods, and geographic areas are considered. Potential contributors may wish to contact the journal before submitting an essay to see if their proposed image and idea for an essay are within the scope and interest of the journal. Contributors must adhere to a number of guidelines and tips for About the Cover submissions:

- Essays are a maximum of 900 words text.
- No abstract or summary line is needed.
- First author biography is not required.
- One high-resolution image is used on the cover of the journal; up to two secondary images might be permitted to accompany the essay.
- Authors are responsible for obtaining any approvals or releases for use of images that are not in the public domain, copyrighted, or otherwise restricted.
- Examples of cover essays may be found in every issue of the journal from 2002 forward (https://wwwnc.cdc.gov/eid/past-covers). Pay attention to format and tone of the essays.
- Provide a caption with information similar to captions of other EID About the Cover essays.
- Essays do not typically include sequentially numbered references but instead use in-text citations and a bibliography.
- Submit the EID Author Checklist (https://wwwnc.cdc.gov/eid/page/author-checklist) along with each essay.
- All essays are peer reviewed, and the editor-in-chief makes the final decision regarding publication.

If you would like to consult about any aspect of an About the Cover essay for upcoming issues of EID, contact the journal at eideditor@cdc.gov. Meanwhile, we hope that many of you are thinking about what you might have written about this month’s cover image, “The Pool of Tears,” and how it could relate to zoonotic infections.
